# Association of Fine Particulate Matter Exposure With Bystander-Witnessed Out-of-Hospital Cardiac Arrest of Cardiac Origin in Japan

**DOI:** 10.1001/jamanetworkopen.2020.3043

**Published:** 2020-04-17

**Authors:** Sunao Kojima, Takehiro Michikawa, Kunihiko Matsui, Hisao Ogawa, Shin Yamazaki, Hiroshi Nitta, Akinori Takami, Kayo Ueda, Yoshio Tahara, Naohiro Yonemoto, Hiroshi Nonogi, Ken Nagao, Takanori Ikeda, Naoki Sato, Hiroyuki Tsutsui

**Affiliations:** 1 Department of General Internal Medicine 3, Kawasaki Medical School General Medical Center, Okayama, Japan; 2 Department of Environmental and Occupational Health, School of Medicine, Toho University, Ota-ku, Tokyo, Japan; 3 Department of General Medicine, Kumamoto University Hospital, Kumamoto, Japan; 4 National Cerebral and Cardiovascular Center, Suita, Japan; 5 Centre for Health and Environmental Risk Research, National Institute for Environmental Studies, Tsukuba, Japan; 6 Centre for Regional Environmental Research, National Institute for Environmental Studies, Tsukuba, Japan; 7 Environmental Health Sciences, Kyoto University Graduate School of Global Environmental Studies, Sakyo-ku, Kyoto, Japan; 8 Department of Cardiovascular Medicine, National Cerebral and Cardiovascular Center, Suita, Japan; 9 Department of Neuropsychopharmacology, National Center of Neurology and Psychiatry, Kodaira, Japan; 10 Intensive Care Center, Shizuoka General Hospital, Shizuoka, Japan; 11 Department of Cardiovascular Center, Nihon University Hospital, Chiyoda-ku, Tokyo, Japan; 12 Department of Cardiovascular Medicine, Toho University Faculty of Medicine, Ota-ku, Tokyo, Japan; 13 Department of Cardiovascular Medicine, Kawaguchi Cardiovascular and Respiratory Hospital, Kawaguchi, Japan; 14 Department of Cardiovascular Medicine, Faculty of Medical Sciences, Kyushu University, Fukuoka, Japan

## Abstract

**Importance:**

Out-of-hospital cardiac arrests (OHCAs) are a major public health concern and a leading cause of death worldwide. Exposure to ambient air pollution is associated with increases in morbidity and mortality and has been recognized as a leading contributor to global disease burden.

**Objective:**

To examine the association between short-term exposure to particulate matter with a diameter of 2.5 μm or smaller (PM_2.5_) and the incidence of OHCAs of cardiac origin and with the development of initial cardiac arrest rhythm.

**Design, Setting, and Participants:**

This case-control study used data from cases registered between January 1, 2005, and December 31, 2016, in the All-Japan Utstein Registry, a prospective, nationwide, population-based database for OHCAs across all 47 Japanese prefectures. These OHCA cases included patients who had bystander-witnessed OHCAs and for whom emergency medical services responders initiated resuscitation before hospital transfer. A case-crossover design was employed for the study analyses. A prefecture-specific, conditional logistic regression model to estimate odds ratios was applied, and a random-effects meta-analysis was used to obtain prefecture-specific pooled estimates. All analyses were performed from May 7, 2019, to January 23, 2020.

**Main Outcomes and Measures:**

The main outcome was the association of short-term PM_2.5_ exposure with the incidence of bystander-witnessed OHCAs of cardiac origin. The differences in the distribution of initial cardiac arrest rhythm in OHCAs among those with exposure to PM_2.5_ were also examined.

**Results:**

In total, 103 189 OHCAs witnessed by bystanders were included in the final analysis. Among the patients who experienced such OHCAs, the mean (SD) age was 75 (15.5) years, and 62 795 (60.9%) were men. Point estimates of the percentage increase for a 10-μg/m^3^ increase in PM_2.5_ at lag0-1 (difference in mean PM_2.5_ concentrations measured on the case day and 1 day before) demonstrated a statistically significantly higher incidence of OHCA across most of the 47 prefectures, without significant heterogeneity (*I*^2^ = 20.1%; *P* = .12). A stratified analysis found an association between PM_2.5_ exposure and OHCAs (% increase, 1.6; 95% CI, 0.1%-3.1%). An initial shockable rhythm, such as ventricular fibrillation or pulseless ventricular tachycardia (% increase, 0.6; 95% CI, –2.0% to 3.2%), was not associated with PM_2.5_ exposure. However, an initial nonshockable rhythm, such as pulseless electrical activity and asystole, was associated with PM_2.5_ exposure (% increase, 1.4; 95% CI, 0.1%-2.7%).

**Conclusions and Relevance:**

Findings from this study suggest that increased PM_2.5_ concentration is associated with bystander-witnessed OHCA of cardiac origin that commonly presents with nonshockable rhythm. The results support measures to reduce PM_2.5_ exposure to prevent OHCAs of cardiac origin.

## Key Points

**Question:**

What is the association of short-term exposure to fine particulate matter (with a diameter of ≤2.5 μm [PM_2.5_]) with bystander-witnessed out-of-hospital cardiac arrests of cardiac origin and with the development of initial cardiac arrest rhythm?

**Findings:**

In this case-control study of 103 189 bystander-witnessed out-of-hospital cardiac arrests, every 10 μg/m^3^ increase in PM_2.5_ levels was associated with a 1.6% increase in out-of-hospital cardiac arrests and with a 1.4% increase in out-of-hospital cardiac arrests initially presenting with a nonshockable rhythm.

**Meaning:**

Results of this study support the implementation of measures to reduce PM_2.5_ exposure for the prevention of out-of-hospital cardiac arrests of cardiac origin.

## Teaser

This case-control study assesses exposure to ambient air pollution, presenting cardiac arrest rhythms, initial resuscitation efforts, and patient and onset factors in individuals who experienced out-of-hospital cardiac arrests in Japan.

## Introduction

Out-of-hospital cardiac arrests (OHCAs) are a major public health concern and a leading cause of death worldwide. More than 350 000 individuals in North America and 275 000 individuals in Europe experience OHCAs annually, and the mean post–cardiac arrest survival rate remains at approximately 10%.^1,2^ The number of people with OHCA has been increasing in Japan, with the most recent reported figure at 110 000,^3^ although the survival rate is comparatively lower than that in Western countries.^4^ In the United States, the 2010 American Heart Association Guidelines for Cardiopulmonary Resuscitation and Emergency Cardiovascular Care outlined comprehensive clinical decision-making rules for the termination of resuscitative efforts and allowed such management before transporting individuals to a hospital if the prescribed parameters were met.^5^ Conversely, in Japan, emergency medical services (EMS) responders cannot make the decision to terminate resuscitation and must continue life-saving efforts until either the return of spontaneous circulation or arrival at the hospital.^4^ Thus, most people who experience OHCA in Japan and are initially treated by EMS personnel are transported to a hospital and are therefore subsequently registered in the All-Japan Utstein Registry. Despite either country’s approach, survival rates have not improved, partially because of the development of nonshockable rather than shockable rhythms as the initial cardiac arrest rhythm.^3,4,6^

Exposure to ambient air pollution increases morbidity and mortality and has been recognized as a leading contributor to the global disease burden.^7^ Air pollution exacerbates existing heart conditions and plays a role in disease pathogenesis, with evidence of adverse effects being stronger for particulate matter than for gaseous pollutants.^8^ Particulate matter with a diameter of 2.5 μm or smaller (PM_2.5_) is composed of elemental carbon, transition metals, complex organic molecules, sulfate, and nitrate.^8^ It can permeate lung alveoli, can enter the bloodstream, and is further absorbed by phagocytes on lung surfaces. Alveolar epithelial cells, in turn, generate oxygen radicals that may trigger inflammatory responses.^8^ This additive inflammatory effect of particulate pollutants may be associated with aggravated existing inflammatory lung diseases and the progression, destabilization, or rupture of atherosclerotic plaques, precipitating acute coronary syndrome.^8^ Exposure to PM_2.5_ is associated with not only increased hospitalization but also a higher risk of death from respiratory and cardiovascular causes.^9^

Both nationwide and multicountry studies have demonstrated that an increase in PM_2.5_ exposure is associated with mortality.^10^ A recent systematic review and meta-analysis found that short-term exposure to PM_2.5_ was associated with elevated OHCA risk.^11^ However, all of the studies included in this meta-analysis were restricted to single cities and involved relatively small numbers of patients who had OHCA.^11^

We conducted a nationwide case-crossover analysis to examine the association between short-term exposure to PM_2.5_ and bystander-witnessed OHCAs of cardiac origin using the All-Japan Utstein Registry, which contains information on all patients with OHCA. In addition, we investigated the differences in the distribution of initial cardiac arrest rhythms in OHCA among patients with exposure to PM_2.5_.

## Methods

### Data Source, Study Area, and EMS System

The All-Japan Utstein Registry is a prospective, nationwide, population-based database for OHCA with Utstein-style data collection.^12^ It was established on January 1, 2005; has been maintained by the Fire and Disaster Management Agency; and has been described in detail elsewhere.^6^ All fire stations with dispatch centers and collaborating medical institutions contribute to the registry. Data from this registry were provided to the Subcommittee on Resuscitation Science of the Japanese Circulation Society in accordance with governmental legal procedures. This study received approval from the Ethics Committee of Kawasaki Medical School, which waived the requirement for patient written informed consent because only deidentified data were used. We followed the Strengthening the Reporting of Observational Studies in Epidemiology (STROBE) reporting guideline.

Japan has an area of approximately 378 000 km^2^, including both urban and rural communities across 47 prefectures from Hokkaido to Okinawa (eTable 1 and eFigure 1 in the Supplement).^13^ In 2018, the total population was approximately 126 million,^14^ and the 728 municipally governed fire stations with dispatch centers throughout the country followed uniform, guideline-based resuscitation protocols.^15^ Japanese EMS personnel are not authorized to terminate resuscitation efforts; most patients who experience an OHCA are transported to the nearest hospital, and their information is entered into the registry. These cases were included in this present study; cases of decapitation, incineration, decomposition, rigor mortis, and dependent cyanosis were excluded.^3,6^

### Study Population

The OHCA cases registered between January 1, 2005, and December 31, 2016, were included in the study. These patients were those who experienced OHCAs that were witnessed by bystanders and for whom EMS responders initiated resuscitation before hospital transfer. Patients whose cardiac arrest occurred after the arrival of EMS responders, who had unwitnessed OHCA, or who had unidentified witness status were excluded. Those whose cardiac arrest occurred during periods when standardized PM_2.5_ data were unavailable (eg, on national holidays) and those with missing PM_2.5_ data were also excluded from the analysis.

Cardiac arrest has been defined as the end of cardiac mechanical activity as indicated by the absence of signs of circulation.^12^ An arrest is presumed to be cardiac in origin unless evidence suggests it is from an external cause (eg, trauma, hanging, drowning, drug overdose, or asphyxiation), a respiratory disease, a cerebrovascular disease, or a malignant tumor, among others. The physicians in charge (mainly emergency department physicians) who interacted with the EMS personnel are responsible for ascertaining the cause of arrest.^3,6^

### Data Collection and Quality Control

Data were collected prospectively according to the Utstein templates for resuscitation registries.^12^ The All-Japan Utstein Registry includes only the name of the prefecture as the place of onset to maintain personal information security. All event times were synchronized with the dispatch center clock.^3,6^ The times of collapse and administration of public access automated external defibrillators were identified through an interview conducted by the EMS personnel with the bystander witness before the EMS personnel left the scene.^3,6^ In cases in which the bystander initiated cardiopulmonary resuscitation, chest compression alone and conventional cardiopulmonary resuscitation with rescue breathing were recorded as presence of bystander resuscitation. The initial cardiac arrest rhythm was classified as shockable (ventricular fibrillation and pulseless ventricular tachycardia) or nonshockable (pulseless electric activity and asystole) based on the electrocardiographic data recorded by the automated external defibrillator.^3,6^ Data forms were completed by EMS personnel in cooperation with the patient’s physician in charge, and the information was subsequently entered into the All-Japan Utstein Registry. Forms were logically checked by the computerized system and were verified by the Implementation Working Group for the All-Japan Utstein Registry. If a data form was incomplete, the Fire and Disaster Management Agency returned it to the respective fire station for completion.^3,6^

### Environmental Data

Hourly measurements of PM_2.5_ concentrations were obtained using automated equipment and standard reference methods from the atmospheric environment database of the National Institute for Environmental Studies in Japan. Although each prefecture had 1 or more ambient air pollution–monitoring station, we incorporated environmental data from only 47 stations that were each located in a distinct prefectural capital, and the measured levels were considered representative of the air quality of that region. We subsequently correlated the PM_2.5_ data as applicable to each patient based on the place (prefecture) of OHCA onset. We checked that the PM_2.5_ concentrations measured at the applicable station correlated with those assessed at other stations in the same prefecture (mean correlation coefficient, 0.9). We collected hourly measurements of PM_2.5_ across a 24-hour period, and we calculated the daily mean concentration values, excluding the days in which more than 4 such values were missing. The median (interquartile range) rate of such missed days in the PM_2.5_ data was 1.5% (0.8%–2.3%).

In addition, we measured the daily mean concentration levels of other pollutants, including nitrogen dioxide and sulfur dioxide. Maximum concentrations of ozone were recorded over 8 hourly periods. Data published by the Japan Meteorological Agency were used to evaluate both the daily mean ambient temperature and relative humidity levels. Incidence of influenza was referenced from the database of the National Institute of Infectious Diseases in Japan. Periods of influenza epidemic were defined as weeks in which the number of recorded cases were greater than the 90th percentile of the distribution during the study period.

### Statistical Analysis

The case-crossover design (a specific type of case-control study) was used to examine the association between short-term exposure to PM_2.5_ and OHCAs of cardiac origin. In a case-crossover study, time-invariant factors, such as age and sex, do not act as confounders because patients are compared with themselves at different periods; that is, a patient’s PM_2.5_ exposure on the case day was compared with the exposure on control days. Although the case day was defined as the day of the OHCA occurrence, control days were selected using a time-stratified method^16^; that is, 3 or 4 control days were chosen from the same day of the week, month, and year as the case day. If a patient experienced a cardiac arrest on June 16, 2016, then June 2, 9, 23, and 30 of that year would be assigned as control days.

This time-stratified approach to the referent selection allowed for unbiased estimations using a conditional logistic regression analysis.^16^ We first applied a prefecture-specific, conditional logistic regression model to estimate the odds ratios (ORs) with 95% CIs for every 10-μg/m^3^ increase in PM_2.5_ concentrations at lag0-1 (difference in mean PM_2.5_ concentrations measured on the case day and 1 day before). The choice of lag0-1 was made before commencing the study and was based on previous evidence.^11,17,18^ Time-variant factors, including ambient temperature at lag0-1 (a 5-knot natural cubic spline), relative humidity at lag0-1 (a 3-knot cubic spline), and incidence of influenza epidemic, were included in the model. Random-effects meta-analysis was used to obtain prefecture-specific pooled estimates of the associations between PM_2.5_ and OHCA. Results were presented as a percentage increase in OHCA incidence, which was calculated as [(OR – 1) × 100]. Heterogeneity was verified using the *I*^2^ statistic. We repeated this analysis for each type of initial cardiac arrest rhythm (shockable or nonshockable).

Furthermore, we constructed a multipollutant model that was adjusted for ozone, nitrogen dioxide, and sulfur dioxide concentrations at lag0-1 to examine the potential confounding effects of different pollutants. We also stratified analyses by age (<75 or ≥75 years) and sex. We performed stratified analyses by season of onset (warm [May to October] or cold [November to April]) and region (East, Central, or West).^10^ We obtained the initial cardiac arrest rhythm of electrocardiogram (ECG), presence of bystander resuscitation, and time from collapse to initial ECG for the association between PM_2.5_ and OHCA to explore the possibility of differences owing to these factors.

All analyses were performed from May 7, 2019, to January 23, 2020, using Stata, version 15 (StataCorp LLC). Analyses used 2-tailed, paired testing. Findings were considered statistically significant at *P* < .05.

## Results

### Patient Characteristics

**Figure 1.**
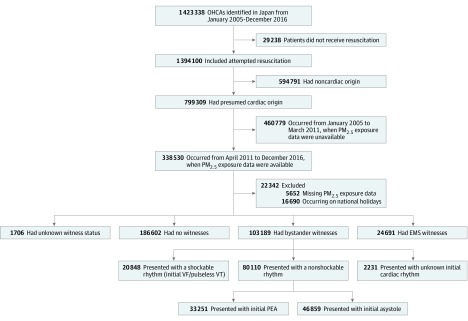
Study Population EMS indicates emergency medical service; OHCA, out-of-hospital cardiac arrest; PEA, pulseless electrical activity; PM_2.5_, particulate matter with a ≤2.5-μm diameter; VF, ventricular fibrillation; and VT, ventricular tachycardia.

A total of 1 423 338 OHCAs were documented in Japan from January 1, 2005, to December 31, 2016. Of this total, 799 309 OHCAs (56.2%) were considered to be of cardiac origin. However, 338 530 OHCAs occurred during the PM_2.5_ monitoring (April 1, 2011, to December 31, 2016), of which 103 189 were witnessed by bystanders. Overall, the initial presentation of 20 848 OHCAs (20.2%) was with shockable rhythm (ventricular fibrillation and pulseless ventricular tachycardia); 80 110 (77.6%) OHCAs presented with nonshockable rhythm, of which 33 251 had pulseless electric activity, 46 859 had asystole, and 2231 (2.2%) had an unknown cardiac rhythm (Figure 1).

Of these 103 189 patients (mean [SD] age, 75 [15.5] years), 63 108 (61.2%) were aged 75 years or older, 62 795 (60.9%) were men, and 46 843 (45.4%) experienced an OHCA during warm seasons. Initial nonshockable rhythm was detected in 80 110 patients who experienced OHCA (77.6%), of whom 54 050 (52.4%) received bystander resuscitation. Time from collapse to initial ECG was less than 10 minutes in 65 770 patients (63.7%) who had OHCA (Table 1).

**Table 1.** Characteristics of Bystander-Witnessed Out-of-Hospital Cardiac Arrest of Cardiac Origin in 47 Prefectures in Japan, April 2011 to December 2016

Characteristic	No. (%)
Total No.	103 189 (100)
Patient age, y	
<75	40 081 (38.8)
≥75	63 108 (61.2)
Patient sex	
Men	62 795 (60.9)
Women	40 394 (39.1)
Season	
Warm (May to October)	46 843 (45.4)
Cold (November to April)	56 346 (54.6)
Region^a^	
East	59 702 (57.9)
Central	28 646 (27.8)
West	14 266 (13.8)
Initial cardiac arrest rhythm of ECG	
Shockable rhythm^b^	20 848 (20.2)
Nonshockable rhythm^c^	80 110 (77.6)
Unknown	2231 (2.2)
Bystander resuscitation	
Present	54 050 (52.4)
Absent	49 045 (47.5)
Unknown	94 (0.1)
Time from collapse to initial ECG, min	
<10	65 770 (63.7)
≥10	37 271 (36.1)
Unknown	148 (0.1)

Abbreviation: ECG, electrocardiogram.

^a^ See eTable 1 and eFigure 1 in the Supplement.

^b^ Indicates ventricular fibrillation and pulseless ventricular tachycardia.

^c^ Indicates pulseless electrical activity and asystole.

The mean (SD) daily PM_2.5_ concentration was 13.9 (7.9) μg/m^3^ according to the nationwide analysis (eTable 2 in the Supplement). Regionally, the mean (SD) PM_2.5_ concentrations were 12.5 (7.4) μg/m^3^ in East Japan, 13.7 (7.8) μg/m^3^ in Central Japan, and 16.3 (8.7) μg/m^3^ in West Japan (eFigure 1 in the Supplement). Prefecture-specific results for environmental factors are presented in eTable 1 in the Supplement. Pearson correlation coefficients for PM_2.5_ concentrations were calculated as *r* = 0.42 for ozone, *r* = 0.31 for nitrogen dioxide, and *r* = 0.44 for sulfur dioxide (eTable 3 in the Supplement).

### PM_2.5_ and Bystander-Witnessed OHCA of Cardiac Origin

**Figure 2.**
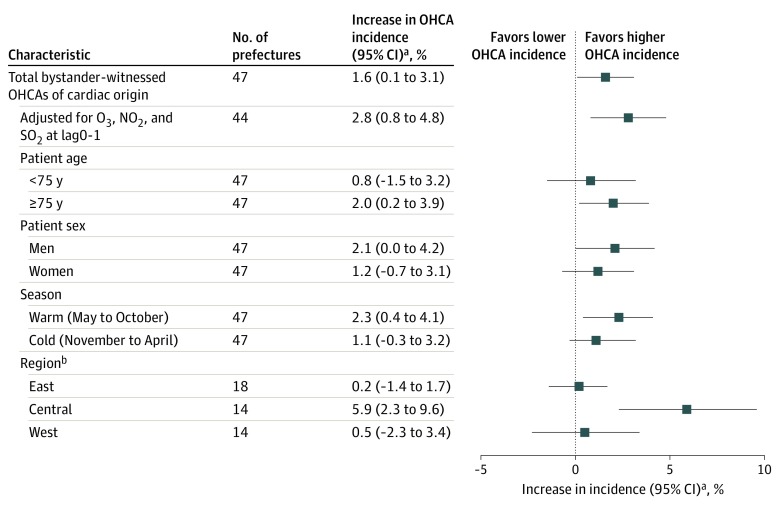
Association Between Exposure to Particulate Matter With Diameter of 2.5-μm or Less (PM_2.5_) and Total Bystander-Witnessed Out-of-Hospital Cardiac Arrest (OHCAs) of Cardiac Origin The OHCAs were adjusted for nitrogen dioxide (NO_2_), ozone (O_3_), and sulfur dioxide (SO_2_), which were adjusted for ambient temperature at lag0-1, relative humidity at lag0-1, and incidence of influenza epidemic. Lag0-1 indicates difference in mean PM_2.5_ concentrations measured on the case day and 1 day before. ^a^Odds ratio for percent increase for every 10-μg/m^3^ increase in PM_2.5_ at lag0-1. ^b^Total number of regions was 46, with Okinawa excluded from the West Japan region because of geographic differences (eTable 1 and eFigure 1 in the Supplement).

On assessing the prefecture-specific associations between the extent of exposure to PM_2.5_ and the occurrence of bystander-witnessed OHCAs with cardiac origin, we found that point estimates of the percentage increase for a 10-μg/m^3^ increase in PM_2.5_ at lag0-1 demonstrated a statistically signifcantly higher incidence of OHCA for most of the 47 prefectures (eFigure 2 in the Supplement) without statistically significant heterogeneity (*I*^2^ = 20.1%; *P* = .12). Figure 2 demonstrates a stratified analysis of the sensitivity of this association (% increase, 1.6; 95% CI, 0.1%-3.1%). After an adjustment for exposure to copollutants (ozone, nitrogen dioxide, and sulfur dioxide), point estimates of the percentage increases tended to be high among these subgroups: 75 years or older (% increase, 2.0; 95% CI, 0.2%-3.9%), men (% increase, 2.1; 95% CI, 0%-4.2%), and warm season onset (% increase, 2.3; 95% CI, 0.4%-4.1%). There was a regional association between PM_2.5_ and OHCA for Central Japan (% increase, 5.9; 95% CI, 2.3%-9.6%) but not for both East Japan (% increase, 0.2; 95% CI, –1.4% to 1.7%) and West Japan (% increase, 0.5; 95% CI, –2.3% to 3.4%).

### PM_2.5_ and Initial Cardiac Arrest Rhythm

Table 2 shows characteristics of bystander-witnessed OHCA of cardiac origin according to the initial cardiac arrest rhythms. Most patients with a nonshockable rhythm were 75 years or older (55 591 [69.4%]), 35 345 were women (44.1%), 44 628 had an onset in the cold season (55.7%), 46 625 lived in East Japan (58.5%), 39 618 did not receive bystander resuscitation (49.5%), and 30 515 had time from collapse to initial ECG of more than 10 minutes (38.2%).

**Table 2.** Characteristics of Bystander-Witnessed OHCA of Cardiac Origin by Initial Cardiac Arrest Rhythms

Characteristic	No. (%)		*P* value
	Shockable rhythm^a^	Nonshockable rhythm^b^	
Total No.	20 848	80 110	
Patient age, y			<.001
<75	14 454 (69.3)	24 519 (30.6)	
≥75	6394 (30.7)	55 591 (69.4)	
Patient sex			<.001
Men	16 604 (79.6)	44 765 (55.9)	
Women	4244 (20.4)	35 345 (44.1)	
Season			<.001
Warm (May to October)	10 284 (49.2)	35 482 (44.3)	
Cold (November to April)	10 564 (50.7)	44 628 (55.7)	
Region^c^			<.001
East	11 661 (56.4)	46 625 (58.5)	
Central	6007 (29.0)	22 104 (27.7)	
West	3027 (14.6)	10 974 (13.8)	
Bystander resuscitation			<.001
Present	12 075 (58.0)	40 446 (50.5)	
Absent	8736 (42.0)	39 618 (49.5)	
Time from collapse to initial ECG, min			<.001
<10	14 689 (70.5)	49 473 (61.8)	
≥10	6138 (29.5)	30 515 (38.2)	

Abbreviation: ECG, electrocardiogram.

^a^ Indicates ventricular fibrillation and pulseless ventricular tachycardia.

^b^ Indicates pulseless electrical activity and asystole.

^c^ See eTable 1 and eFigure 1 in the Supplement.

**Figure 3.**
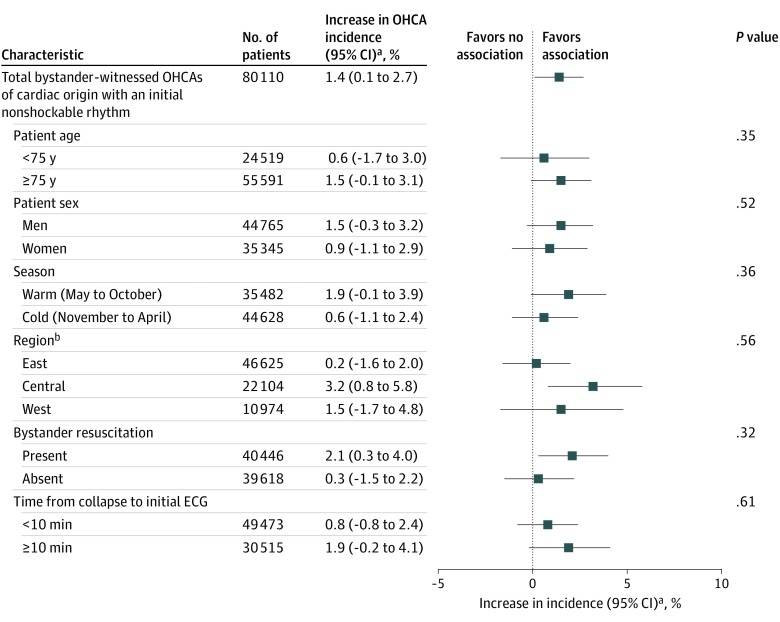
Stratified Analyses Comparing Exposure to Particulate Matter With Diameter of 2.5 μm or Less (PM_2.5_) and Bystander-Witnessed Out-of-Hospital Cardiac Arrest (OHCAs) of Cardiac Origin With an Initial Nonshockable Rhythm Ambient temperature at lag0-1, relative humidity at lag0-1, and incidence of influenza epidemic were included in the model. ECG indicates electrocardiogram; lag0-1 indicates difference in mean PM_2.5 _concentrations measured on the case day and 1 day before. ^a^Odds ratio for percent increase for every 10-μg/m^3^ increase in PM_2.5_ at lag0-1. ^b^Total number of regions was 46, with Okinawa excluded from the West Japan region because of geographic differences (eTable 1 and eFigure 1 in the Supplement).

We analyzed the respective percentage increases for the association of PM_2.5_ exposure with OHCA according to the distribution of presenting cardiac arrest rhythm. Although the initial shockable rhythm (% increase, 0.6; 95% CI, –2.0% to 3.2%) was not associated with the PM_2.5_ exposure, the nonshockable rhythm showed statistically significant association (% increase, 1.4; 95% CI, 0.1%-2.7%). In the stratified analyses, the initial nonshockable rhythm in Central Japan (% increase, 3.2; 95% CI, 0.8%-5.8%) and presence of bystander resuscitation (% increase, 2.1; 95% CI, 0.3%-4.0%) were associated with PM_2.5_ exposure. However, we did not observe a statistically significant difference in the development of nonshockable rhythm among bystander-witnessed OHCAs of cardiac origin according to age, sex, season of onset, time from collapse to initial ECG, region, or bystander resuscitation (Figure 3).

## Discussion

Findings of this study suggest that short-term exposure to particulate pollutants is associated with bystander-witnessed OHCAs of cardiac origin in Japan. Increased OHCA incidence was associated with the mean increase in PM_2.5_ concentrations greater than those levels observed on the day before the cardiac arrest. Exposure to PM_2.5_ was also associated with nonshockable rhythm as the initial, presenting OHCA rhythm.

Air pollution has been reported to play a role in the development of cardiovascular diseases.^8^ Previous studies have observed an association between elevated pollutant concentration and risk of myocardial infarction^19^ and an approximately 1.0% worldwide increase in the mean all-cause mortality for every incremental 10-μg/m^3^ short-term exposure to PM_2.5_.^8,10,20^

In a meta-analysis of 12 studies, the short-term exposure to PM_2.5_ was associated with an increased risk of OHCA; however, those studies were performed mainly in a single city.^11^ Results of a study of more than 20 000 Asian individuals with OHCA corroborate our finding that an increase in OHCA incidence and risk may be associated with PM_2.5_ exposure.^21^ Nonetheless, the present study has advantages over other studies owing to the larger sample size (nationwide data of more than 100 000 bystander-witnessed OHCAs of cardiac origin were analyzed) and the random-effects meta-analysis used to obtain prefecture-specific pooled estimates of the association between PM_2.5_ and OHCA, which may yield higher accuracy. Furthermore, we studied the implications of novel factors, including seasonal and regional differences, for the association between PM_2.5_ exposure and OHCAs, along with the types of presenting cardiac arrest rhythms.

As in past studies, we found that older individuals (aged ≥75 years) and men experienced more OHCAs associated with PM_2.5_ exposure, and this association may reflect the frequency of occurrence of cardiovascular disease in these populations.^17,21^ A significant association between PM_2.5_ and OHCA of cardiac origin was observed during warm seasons and in Central Japan, which may indicate seasonal and regional variations in the PM_2.5_ chemical compositions. Nitrate concentrations have been demonstrated to vary by season^22^ and to be low in the summer. Conversely, although sulfate concentrations are high in the summer,^22^ levels in Central Japan are lower than those in West Japan, which is susceptible to the effects of long-range transport of air pollutants.^23^ However, seasonal and regional differences in nitrate and sulfate concentrations did not correlate with the association between PM_2.5_ and OHCA of cardiac origin across the applicable seasons and regions. Little evidence is available on the implications of PM_2.5_ composition for health in Japan. Therefore, further research is required to assess the seasonal and regional differences of particulate air pollutants and their implications for health.

Interest in the association between air pollution and cardiovascular disease occurrence has been growing, most notably interest in the association between PM_2.5_ and cardiac arrest.^8^ Previous studies found inconsistent evidence supporting an association of PM_2.5_ exposure with ventricular tachyarrhythmias in patients with implantable cardioverter defibrillators.^24,25,26^ However, a few studies that described an association between asystole or a nonshockable initial OHCA rhythm with an increase in PM_2.5_ exposure have received less attention.^21,27^ In the present study, the occurrence of an initial nonshockable cardiac rhythm was associated with an increase in PM_2.5_ concentration. Although the pathophysiological details of this effect have not been elucidated, individuals with traditional risk factors are likely to be at a higher risk for cardiovascular events because of exposure to air pollutants.^19,20^ Bystander-witnessed OHCAs of cardiac origin occurring secondary to heterogeneous underlying diseases may be accompanied by higher risks after PM_2.5_ exposure, including susceptibility to presenting with nonshockable rhythm. Older age, female sex, absence of bystander resuscitation, and a longer time from collapse to initial ECG interval may be associated with the development of initial nonshockable rhythm.^28^ This association was also observed in comparisons of the occurrence of shockable rhythms in this study. However, the association between PM_2.5_ exposure and the development of nonshockable rhythm may not be modifiable by these factors because of the lack of adequate study power. Therefore, it would be premature to explain why we observed the association between increased PM_2.5_ concentration and a presentation with a nonshockable rhythm. Nevertheless, the All-Japan Utstein Registry data have demonstrated the current nationwide status of OHCAs in Japan because almost all Japanese patients who experienced a cardiac arrest were registered.^3,4,6^

### Limitations

This study has several limitations. First, similar to past epidemiologic environmental studies of the health effects of air pollution, this study demonstrated that the occurrence of exposure misclassification was inevitable given that the results were based on pollutant data measured at a single monitoring station in each prefecture. It was difficult to estimate the biasing effect of a combination of the Berkson error and classical error on our estimates of the association between PM_2.5_ and OHCA.^29^ Second, as in all epidemiologic studies, the data integrity, validity, and ascertainment bias in this study were potential limitations. However, the uniform data collection, large sample size, and implementation of a population-based design that covered all known OHCAs in Japan may have minimized the effects of these potential sources of bias.^3,4,6^

## Conclusions

Increased PM_2.5_ concentration appeared to be associated with bystander-witnessed OHCAs of cardiac origin that commonly present with a nonshockable rhythm. The results of this case-control study may support the implementation of measures to reduce PM_2.5_ exposure for the prevention of OHCAs with cardiac origin.

## Notes

Supplement. **eTable 1.** Descriptive Data on the Study Period of Bystander Witnessed Out-of-Hospital Cardiac Arrest of Cardiac Origin and Environmental Factors for 47 Prefectures in Japan **eTable 2.** Overall Statistics of Environmental Factors in 47 Prefectures in Japan, from April 2011 to December 2016 **eTable 3.** Pearson Correlation Coefficients Among Daily Mean Pollutant Concentrations **eFigure 1.** Location of 47 Japanese Prefectures and Daily Mean PM_2.5_ Concentrations **eFigure 2.** Prefecture-Specific Odds Ratios of Bystander-Witnessed Out-of-Hospital Cardiac Arrest of Cardiac Origin Incidence With a 10 μg/m^3^ increase in PM_2.5_ Concentrations at lag0-1
