# Supplementary material for: Association of Fine Particulate Matter Exposure With Bystander-Witnessed Out-of-Hospital Cardiac Arrest of Cardiac Origin in Japan
Source: JAMA Netw Open. 2020 Apr 17;3(4):e203043. doi: 10.1001/jamanetworkopen.2020.3043 (PMC7165302; doi:10.1001/jamanetworkopen.2020.3043)
Supplement: Supplement. — eTable 1. Descriptive Data on the Study Period of Bystander Witnessed Out-of-Hospital Cardiac Arrest of Cardiac Origin and Environmental Factors for 47 Prefectures in Japan eTable 2. Overall Statistics of Environmental Factors in 47 Prefectures in Japan, from April 2011 to December 2016 eTable 3. Pearson Correlation Coefficients Among Daily Mean Pollutant Concentrations eFigure 1. Location of 47 Japanese Prefectures and Daily Mean PM2.5 Concentrations eFigure 2. Prefecture-Specific Odds Ratios of Bystander-Witnessed Out-of-Hospital Cardiac Arrest of Cardiac Origin Incidence With a 10 μg/m3 increase in PM2.5 Concentrations at lag0-1 [file jamanetwopen-3-e203043-s001.pdf]

Supplementary Online Content

Kojima S, Michikawa T, Matsui K, et al; the Japanese Circulation Society With Resuscitation Science Study (JCS-ReSS) Group. Association of fine particulate matter exposure with bystander-witnessed out-of-hospital cardiac arrest of cardiac origin in Japan. *JAMA Netw Open.* 2020;3(4):e203043. doi:10.1001/jamanetworkopen.2020.3043

eTable 1. Descriptive Data on the Study Period of Bystander Witnessed Out-of-Hospital Cardiac Arrest of Cardiac Origin and Environmental Factors for 47 Prefectures in Japan

eTable 2. Overall Statistics of Environmental Factors in 47 Prefectures in Japan, from April 2011 to December 2016

eTable 3. Pearson's Correlation Coefficients Among Daily Mean Pollutant Concentrations

eFigure 1. Location of 47 Japanese Prefectures and Daily Mean $PM_{2.5}$ Concentrations

eFigure 2. Prefecture-Specific Odds Ratios of Bystander-Witnessed Out-of-Hospital Cardiac Arrest of Cardiac Origin Incidence With a 10 $\mu g/m^3$ increase in $PM_{2.5}$ Concentrations at lag0-1

This supplementary material has been provided by the authors to give readers additional information about their work.

©2020 Kojima S et al. *JAMA Network Open*

eTable 1. Descriptive Data on the Study Period of Bystander Witnessed Out-of-Hospital Cardiac Arrest of Cardiac Origin and Environmental Factors for 47 Prefectures in Japan

						$PM_{2.5}$ (μg/m^3)						O_3 (ppb)			NO_2 (ppb)			SO_2(ppb)			Ambient temperature (ºC)			Relative humidity (%)		
No.	Prefecture	Region	Study period		Days	n	Mean	SD	25th	Median	75th	n	Mean	SD	n	Mean	SD	n	Mean	SD	n	Mean	SD	n	Mean	SD
1	Hokkaido	East	Apr-12	Dec-16	1736	1,726	7.9	5.7	4.3	6.7	10.4	977	41.5	11.4	1,734	9.6	7.2	1,736	0.6	0.9	1,736	10.1	9.6	1,736	68	11
2	Aomori	East	Apr-13	Dec-16	1371	1,363	11.5	6.6	7.2	10.2	14.0				1,371	11.7	8.8				1,371	11.7	8.8	1,367	75	11
3	Iwate	East	Apr-11	Dec-16	2102	2,070	12.9	6.6	8.1	11.7	16.5	2,024	37.5	12.0	2,077	8.4	5.5	2,077	0.4	0.5	2,102	11.4	9.5	2,099	74	11
4	Miyagi	East	Apr-11	Dec-16	2102	2,063	12.2	7.5	6.9	10.8	16.1	2,057	39.3	12.1	1,712	10.4	5.3	2,071	0.5	0.5	2,102	13.5	8.5	2,102	70	13
5	Akita	East	Apr-12	Dec-16	1736	1,718	13.2	7.7	7.9	11.0	16.3	1,725	44.5	11.0	1,720	4.6	2.6	1,731	0.9	0.7	1,736	12.9	9.1	1,736	73	11
6	Yamagata	East	Apr-12	Dec-16	1736	1,719	11.7	7.4	6.4	10.0	15.6	1,698	42.2	13.3	1,675	9.6	6.1	1,700	1.1	0.8	1,736	12.9	9.4	1,736	72	12
7	Fukushima	East	Apr-14	Dec-16	1006	996	10.5	6.3	5.8	9.2	14.0	994	40.2	12.7	997	8.1	4.9	1,001	0.5	0.5	1,006	14.9	8.4	1,006	70	14
8	Ibaraki	East	Apr-12	Dec-16	1736	1,701	11.5	6.7	6.7	10.0	14.4	1,708	42.5	13.4	1,712	7.6	3.2	1,721	0.6	0.4	1,736	15.0	8.1	1,736	73	13
9	Tochigi	East	Apr-13	Dec-16	1371	1,364	12.9	7.1	7.9	11.5	16.6	1,359	46.0	17.9	1,356	10.4	4.8				1,371	15.3	8.3	1,371	68	14
10	Gunma	East	Apr-11	Dec-16	2102	2,067	14.2	9.0	7.5	13.2	19.5	2,075	47.1	16.9	2,092	7.6	3.9	2,050	1.4	0.7	2,102	15.7	8.6	2,102	61	15
11	Saitama	East	Apr-13	Dec-16	1371	1,363	13.3	7.5	7.9	11.8	17.0	1,363	42.3	17.4	1,328	15.1	7.1	1,365	0.8	0.5	1,371	16.4	8.2	1,371	64	16
12	Chiba	East	Apr-12	Dec-16	1736	1,713	14.4	9.6	7.8	12.1	18.6	1,725	39.8	15.2	1,729	11.1	6.3	1,733	1.7	1.1	1,736	17.0	7.6	1,736	66	16
13	Tokyo	East	Apr-11	Dec-16	2102	2,060	16.6	8.6	10.5	14.8	20.7	2,070	35.2	14.7	2,081	22.8	9.2	2,087	2.0	1.1	2,102	17.0	8.0	2,102	64	16
14	Kanagawa	East	Apr-12	Dec-16	1736	1,718	14.3	8.2	8.5	12.6	18.3	1,722	38.1	15.7	1,715	17.8	8.3	1,674	3.8	2.7	1,736	17.0	7.6	1,721	68	16
15	Niigata	East	Apr-12	Dec-16	1736	1,705	11.9	7.7	6.7	10.3	15.0	1,624	45.7	12.1	1,677	8.5	4.1	1,660	1.4	0.8	1,736	14.7	8.7	1,736	73	10
16	Toyama	Central	Apr-12	Dec-16	1736	1,730	10.5	7.2	5.6	9.0	13.8	1,678	46.1	12.3	1,730	7.8	3.9	1,734	0.8	0.5	1,736	15.3	8.8	1,736	73	12
17	Ishikawa	Central	Apr-13	Dec-16	1371	1,350	11.7	7.1	6.8	9.9	14.9	1,355	44.6	13.3	1,363	8.7	4.0	1,367	1.4	1.6	1,371	16.0	8.4	1,371	70	11
18	Fukui	Central	Apr-11	Dec-16	2102	2,032	14.5	8.4	8.5	12.9	18.7	2,019	43.7	14.5	2,025	7.8	3.9	2,048	1.1	0.7	2,102	15.5	8.9	2,102	74	11
19	Yamanashi	East	Apr-11	Dec-16	2102	2,078	12.1	7.5	6.9	10.6	15.9	2,055	42.9	14.9	2,081	11.5	6.3	2,079	1.5	0.8	2,102	15.7	8.8	2,102	63	14
20	Nagano	East	Apr-11	Dec-16	2102	1,985	11.4	6.8	6.3	10.1	15.2	2,040	44.3	14.2	2,072	5.6	4.5	2,076	2.7	2.1	2,102	13.0	9.5	2,102	73	11
21	Gifu	Central	Apr-12	Dec-16	1736	1,678	12.7	7.6	7.2	11.6	16.6	1,713	44.9	15.0	1,686	8.9	3.7	1,728	0.7	0.7	1,736	16.9	8.5	1,736	66	13
22	Shizuoka	East	Apr-13	Dec-16	1371	1,348	12.3	7.2	7.3	10.7	15.2	1,312	44.0	14.9	1,363	9.5	3.6	1,293	1.3	0.4	1,371	17.7	7.2	1,371	68	14

©2020 Kojima S et al. *JAMA Network Open*

23	Aichi	Central	Apr-11	Dec-16	2102	2,055	15.7	8.9	9.3	14.1	20.1	2,052	43.1	15.7	2,063	15.1	7.6	2,067	1.6	0.8	2,102	16.8	8.5	2,102	66	13
24	Mie	Central	Apr-12	Dec-16	1736	1,722	12.8	7.6	7.2	11.5	16.5	1,717	46.8	14.7	1,726	7.2	4.4	1,729	1.4	0.6	1,736	17.0	8.1	1,736	67	13
25	Shiga	Central	Apr-13	Dec-16	1371	1,354	12.0	7.4	6.6	10.5	15.9	1,339	46.4	13.7	1,354	7.0	4.0				1,371	16.1	8.3	1,371	72	10
26	Kyoto	Central	Apr-12	Dec-16	1736	1,700	13.6	7.3	8.4	12.1	17.2	1,592	43.5	15.0	1,625	11.8	5.6	1,730	3.6	1.1	1,736	17.0	8.6	1,736	66	10
27	Osaka	Central	Apr-12	Dec-16	1736	1,721	17.1	8.9	10.5	15.3	21.4	1,680	35.9	14.9	1,708	23.3	9.1	1,675	4.3	2.1	1,736	17.7	8.2	1,736	64	11
28	Hyogo	Central	Apr-13	Dec-16	1371	1,361	14.5	8.0	8.8	12.8	18.5	1,352	40.8	14.8	936	15.8	7.7	1,358	2.8	1.8	1,371	17.9	7.9	1,371	64	11
29	Nara	Central	Apr-12	Dec-16	1736	1,706	12.8	7.8	7.0	11.2	16.7	1,637	44.0	15.6	1,701	9.2	4.8	1,695	3.5	1.3	1,736	15.9	8.4	1,736	74	11
30	Wakayama	Central	Apr-12	Dec-16	1736	1,724	15.2	7.8	9.8	14.0	19.1	1,720	43.7	14.9	1,719	8.7	3.6	1,723	2.5	1.2	1,736	17.6	8.0	1,736	67	11
31	Tottori	West	Apr-11	Dec-16	2102	2,053	16.0	8.6	9.9	14.2	20.2	2,063	44.7	13.0	2,082	3.7	2.3	2,088	0.5	0.9	2,102	15.8	8.5	2,101	73	10
32	Shimane	West	Apr-13	Dec-16	1371	1,331	14.0	7.8	8.3	12.2	18.3	1,300	48.2	14.1	1,245	2.7	1.4	362	0.7	0.7	1,371	16.1	8.0	1,370	77	10
33	Okayama	West	Apr-12	Dec-16	1736	1,728	17.6	9.1	11.1	15.6	22.4	1,723	42.7	15.8	1,718	11.5	4.9	1,736	4.9	1.6	1,736	16.9	8.4	1,736	68	12
34	Hiroshima	West	Apr-11	Dec-16	2102	2,035	18.2	8.5	11.8	16.7	23.0	2,000	43.6	16.2	2,029	13.3	6.2	2,090	1.1	0.8	2,102	17.0	8.3	2,102	66	11
35	Yamaguchi	West	Jun-11	Dec-16	2041	1,925	14.6	8.3	8.4	13.1	19.1	2,009	41.1	15.1	2,024	8.7	3.9	2,027	0.9	0.7	2,041	16.3	8.5	2,041	74	10
36	Tokushima	Central	Apr-11	Dec-16	2102	2,018	13.9	8.3	7.9	12.2	17.8	2,056	43.3	15.9	1,918	9.5	4.4	2,089	1.3	0.8	2,102	17.3	7.9	2,102	69	12
37	Kagawa	West	Apr-12	Dec-16	1736	1,716	17.9	9.6	11.0	16.0	22.8	1,664	39.7	13.8	1,728	14.4	8.0	1,732	6.4	3.3	1,736	17.4	8.2	1,736	67	12
38	Ehime	West	Apr-12	Dec-16	1736	1,723	16.9	8.4	10.5	15.5	21.4	1,657	39.9	13.5	1,690	10.5	4.5	1,709	2.8	2.1	1,736	17.4	7.9	1,736	69	12
39	Kochi	Central	Apr-12	Dec-16	1736	1,676	14.5	7.5	9.2	12.7	18.2	1,597	42.6	13.7	1,695	5.5	3.1	1,682	3.4	1.1	1,736	17.9	7.6	1,733	70	14
40	Fukuoka	West	Apr-12	Dec-16	1736	1,722	18.2	8.9	11.5	16.7	22.8	1,716	40.6	14.3	1,724	16.3	5.3	1,728	2.5	1.5	1,736	18.0	7.7	1,736	70	13
41	Saga	West	Apr-12	Dec-16	1736	1,712	14.8	8.8	8.3	13.3	19.7	1,717	44.5	14.7	1,727	8.1	4.1	1,716	2.1	1.2	1,736	17.6	8.0	1,736	72	11
42	Nagasaki	West	Apr-14	Dec-16	1006	1,002	15.9	8.3	9.7	14.6	20.3	987	39.8	13.1	993	6.5	3.0	995	1.6	1.0	1,006	18.2	7.1	1,006	74	12
43	Kumamoto	West	Apr-13	Mar-16	1096	1,080	18.4	10.0	10.8	17.2	24.0	930	44.2	15.3	1,072	8.3	4.5	1,089	2.6	2.2	1,096	17.1	8.0	1,096	72	12
44	Oita	West	Apr-12	Dec-16	1736	1,700	15.3	8.6	8.7	13.7	19.8	1,354	43.0	14.5	1,362	6.9	3.0	1,370	2.4	1.3	1,736	17.3	7.6	1,736	71	12
45	Miyazaki	West	May-13	Dec-16	1341	1,293	13.1	8.0	7.5	11.6	17.3	1,328	32.2	12.9	886	4.4	2.3	1,331	3.8	2.1	1,341	18.5	7.3	1,341	76	12
46	Kagoshima	West	Apr-12	Dec-16	1736	1,676	17.0	8.3	10.9	15.6	21.7	1,640	40.8	13.1	1,683	5.8	3.7	1,671	2.0	4.1	1,736	19.3	7.2	1,736	73	11
47	Okinawa	–	Apr-14	Dec-16	1006	942	9.9	5.8	5.7	8.4	12.3	965	34.5	15.6	981	6.7	3.4	963	0.4	0.6	1,006	24.2	4.7	1,006	74	11

NO_2, nitrogen dioxide; O_3, ozone; $PM_{2.5}$, particulate matter with an aerodynamic diameter ≤2.5 µm; ppb, parts per billion; SD, standard deviation; SO_2, sulphur dioxide

©2020 Kojima S et al. *JAMA Network Open*

eTable 2. Overall Statistics of Environmental Factors in 47 Prefectures in Japan, from April 2011 to December 2016

Environmental Factors (Daily Mean)	Number of Prefectures	Mean (SD)	Percentile		
			25th	50th	75th
$PM_{2.5}$ (µg/m^3)	47	13.9 (7.9)	8.2	12.4	17.9
O_3 (ppb)*	46	42.2 (14.3)	32.3	40.6	51.2
NO_2 (ppb)	47	9.9 (4.9)	6.3	8.9	12.5
SO_2 (ppb)	44	1.9 (1.2)	1.1	1.6	2.4
Ambient temperature (°C)	47	16.3 (8.2)	8.9	17.0	23.2
Relative humidity (%)	47	70 (12)	62	70	78

NO_2, nitrogen dioxide; O_3, ozone; $PM_{2.5}$, particulate matter with an aerodynamic diameter ≤2.5 µm; ppb, parts per billion; SD, standard deviation; SO_2, sulphur dioxide

*Daily maximum 8-h mean concentrations

©2020 Kojima S et al. *JAMA Network Open*

eTable 3. Pearson's Correlation Coefficients Among Daily Mean Pollutant Concentrations

	$PM_{2.5}$ (µg/m^3)	O_3 (ppb)*	NO_2 (ppb)	SO_2 (ppb)
$PM_{2.5}$ (µg/m^3)	1			
O_3 (ppb)*	0.42	1		
NO_2 (ppb)	0.31	-0.22	1	
SO_2 (ppb)	0.44	0.10	0.34	1

NO_2, nitrogen dioxide; O_3, ozone; $PM_{2.5}$, particulate matter with an aerodynamic diameter ≤2.5 µm; ppb, parts per billion; SO_2, sulphur dioxide

*Daily maximum 8-h mean concentrations

©2020 Kojima S et al. *JAMA Network Open*

eFigure 1. Location of 47 Japanese Prefectures and Daily Mean $PM_{2.5}$ Concentrations

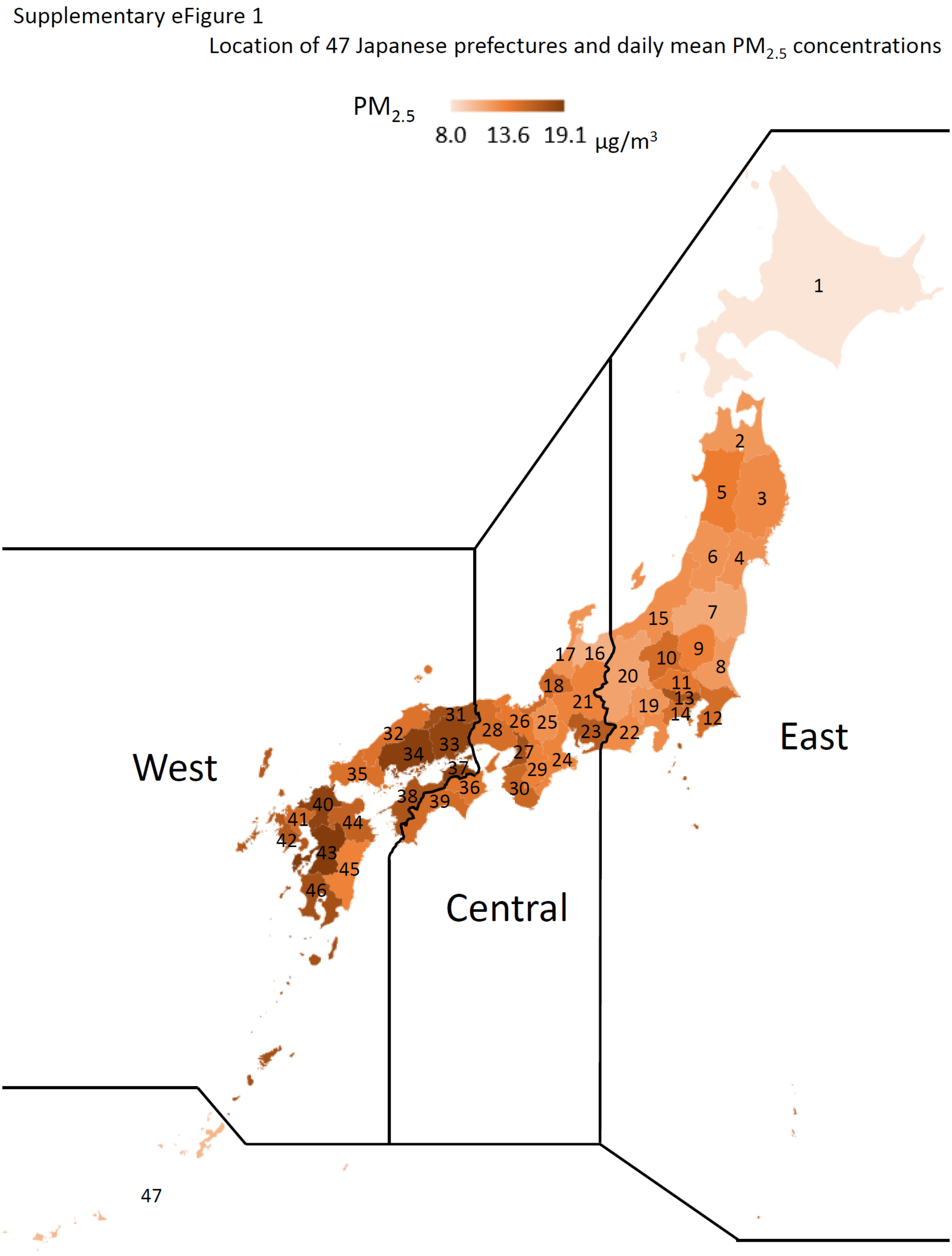

©2020 Kojima S et al. *JAMA Network Open*

eFigure 2. Prefecture-Specific Odds Ratios of Bystander-Witnessed Out-of-Hospital Cardiac Arrest of Cardiac Origin Incidence With a 10 μg/m^3 increase in $PM_{2.5}$ Concentrations at lag0-1

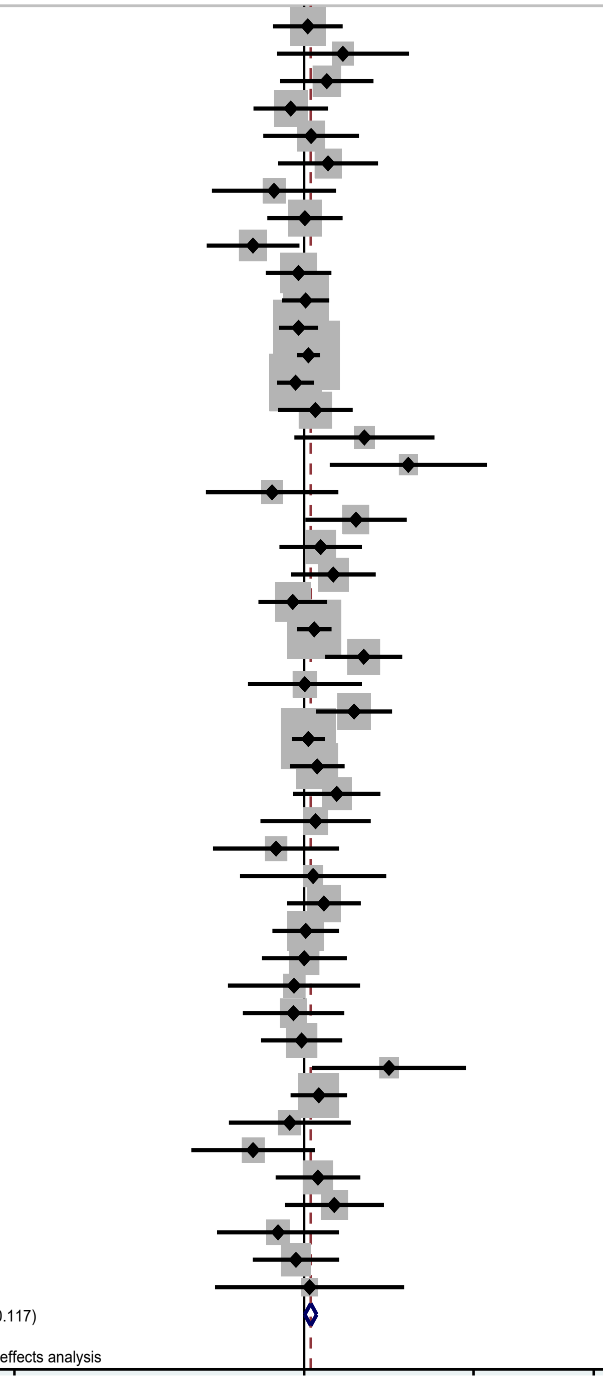

number	prefecture	OR (95% CI)	% Weight
1	Hokkaido	1.01 (0.93, 1.10)	2.41
2	Aomori	1.10 (0.94, 1.29)	0.78
3	Iwate	1.06 (0.94, 1.18)	1.47
4	Miyagi	0.97 (0.89, 1.06)	2.16
5	Akita	1.02 (0.91, 1.14)	1.41
6	Yamagata	1.06 (0.94, 1.19)	1.31
7	Fukushima	0.93 (0.80, 1.08)	0.87
8	Ibaraki	1.00 (0.92, 1.10)	2.13
9	Tochigi	0.88 (0.79, 0.99)	1.49
10	Gunma	0.99 (0.91, 1.07)	2.65
11	Saitama	1.00 (0.95, 1.06)	4.30
12	Chiba	0.99 (0.94, 1.03)	5.42
13	Tokyo	1.01 (0.98, 1.04)	8.58
14	Kanagawa	0.98 (0.94, 1.02)	5.76
15	Niigata	1.03 (0.94, 1.12)	2.17
16	Toyama	1.16 (0.98, 1.37)	0.70
17	Ishikawa	1.28 (1.06, 1.55)	0.56
18	Fukui	0.93 (0.79, 1.09)	0.78
19	Yamanashi	1.13 (1.00, 1.28)	1.27
20	Nagano	1.04 (0.94, 1.15)	1.83
21	Gifu	1.07 (0.97, 1.19)	1.75
22	Shizuoka	0.97 (0.90, 1.06)	2.47
23	Aichi	1.02 (0.98, 1.07)	6.18
24	Mie	1.15 (1.05, 1.26)	2.05
25	Shiga	1.00 (0.87, 1.15)	1.03
26	Kyoto	1.13 (1.03, 1.23)	2.10
27	Osaka	1.01 (0.97, 1.05)	6.44
28	Hyogo	1.03 (0.97, 1.10)	3.50
29	Nara	1.08 (0.97, 1.20)	1.65
30	Wakayama	1.03 (0.90, 1.17)	1.09
31	Tottori	0.94 (0.80, 1.09)	0.85
32	Shimane	1.02 (0.86, 1.22)	0.64
33	Okayama	1.05 (0.96, 1.15)	2.21
34	Hiroshima	1.00 (0.93, 1.09)	2.59
35	Yamaguchi	1.00 (0.90, 1.11)	1.73
36	Tokushima	0.98 (0.83, 1.14)	0.78
37	Kagawa	0.97 (0.86, 1.10)	1.27
38	Ehime	0.99 (0.90, 1.10)	1.87
39	Kochi	1.23 (1.02, 1.47)	0.59
40	Fukuoka	1.04 (0.97, 1.11)	3.33
41	Saga	0.97 (0.83, 1.12)	0.91
42	Nagasaki	0.89 (0.76, 1.03)	0.89
43	Kumamoto	1.03 (0.93, 1.14)	1.74
44	Oita	1.08 (0.95, 1.21)	1.33
45	Miyazaki	0.94 (0.81, 1.09)	0.91
46	Kagoshima	0.98 (0.88, 1.09)	1.68
47	Okinawa	1.01 (0.81, 1.27)	0.39
Overall (I-squared = 20.1%, p = 0.117)		1.02 (1.00, 1.03)	100.00

NOTE: Weights are from random effects analysis

©2020 Kojima S et al. *JAMA Network Open*
